# Potential health benefits of bromelain: a critical review of the current literature

**DOI:** 10.3389/fnut.2026.1744666

**Published:** 2026-05-20

**Authors:** Nevin Sanlier, Esra Irmak, Erva Ankarali

**Affiliations:** Department of Nutrition and Dietetics, Faculty of Health Sciences, Ankara Medipol University, Ankara, Türkiye

**Keywords:** anti-inflammatory, antioxidant, bromelain, obesity, pineapple

## Abstract

This review summarizes current knowledge on bromelain, a proteolytic enzyme complex derived from *Ananas comosus* (pineapple), with a focus on its structure, absorption, metabolism, and therapeutic mechanisms. Bromelain exhibits a wide range of biological activities, including antioxidative, anti-inflammatory, immunomodulatory, antinociceptive, antiulcerative, and antihyperlipidemic effects. These effects are primarily mediated through modulation of key inflammatory and oxidative stress pathways, including inhibition of NF-κB signaling via suppression of IκBα degradation, downregulation of MAPK pathways (ERK, JNK, p38), and activation of antioxidant responses through Nrf2 signaling. Emerging evidence suggests that bromelain may have therapeutic potential in cancer, cardiovascular, metabolic, infectious, and neurodegenerative diseases; however, most findings are derived from *in vitro* and animal studies, with limited clinical evidence. Its efficacy is influenced by its bioavailability, which may be affected by gastrointestinal degradation, although advanced formulation approaches such as enzyme stabilization and delivery systems have been proposed to enhance its systemic activity. While current data support bromelain as a promising adjunct in disease management, further well-designed clinical studies are required to establish optimal dosing, long-term safety, and clinical efficacy.

## Graphical abstract

**Figure:**
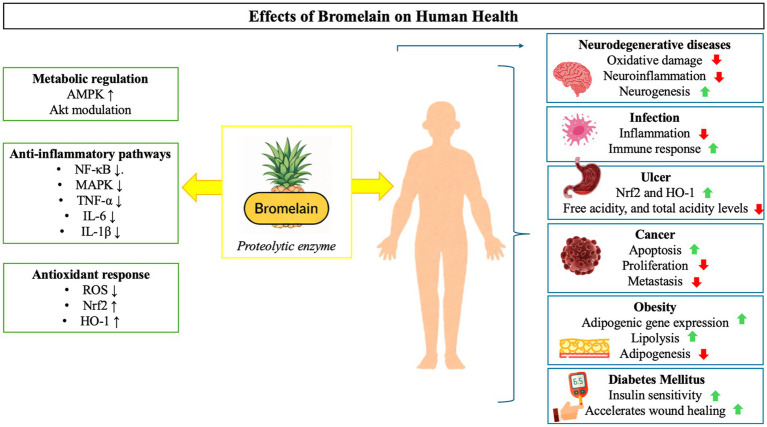

## 1 Introduction

Pineapple (*Ananas comosus* (L.) Merr.), a member of the Bromeliaceae family, is widely cultivated in tropical and subtropical regions and consumed globally in both fresh and processed forms (1). However, only about 25% of the plant is utilized in the food industry, while the remaining 75%, including the peel, core, stem, and leaves, is considered agricultural waste (2). Approximately 35.5% of the fruit consists of peel and 14.7% of core (3). Although pineapple contains various nutritional components such as natural sugars, amino acids, polyphenols, organic acids, and essential micronutrients (4, 5), increasing scientific attention has focused on bromelain, a bioactive proteolytic enzyme complex considered one of its most important bioactive constituents. It is important to clearly distinguish bromelain from other bioactive constituents of pineapple, such as polyphenols, vitamins, and organic acids, as these compounds contribute differently to biological activity and operate through distinct molecular mechanisms (6, 7).

Bromelain is primarily obtained from the stem and fruit tissues of pineapple, with higher concentrations found in the stem (2, 4). It has attracted considerable scientific interest due to its broad spectrum of biological activities. A growing body of evidence indicates that bromelain exerts anti-inflammatory, antioxidant, immunomodulatory, and antiproliferative effects through multiple molecular mechanisms, including the downregulation of pro-inflammatory cytokines such as tumor necrosis factor-α (TNF-α) and interleukin-6 (IL-6), inhibition of key inflammatory signaling pathways such as nuclear factor kappa B (NF-κB), and modulation of immune cell activation and migration (2, 6, 7).

This review aims to provide a comprehensive overview of bromelain, focusing on its chemical structure, metabolism, and its broad spectrum of biological activities, including antioxidant, anti-inflammatory, immunomodulatory, antiulcer, antiobesity, and antihyperlipidemic effects. In addition, its potential roles in disease prevention and management are discussed, along with future research perspectives.

## 2 Method

A comprehensive literature review was conducted to evaluate the biological activities and health-related effects of bromelain. Electronic databases including PubMed, ScienceDirect, Scopus, Google Scholar, Cochrane Library, EMBASE, and Web of Science were systematically searched. The search strategy was developed using combinations of the following keywords and Medical Subject Headings (MeSH): “bromelain,” “*Ananas comosus*,” “proteolytic enzyme,” “bioactive compounds,” “antioxidant,” “anti-inflammatory,” “immunomodulatory,” “anticancer,” “antidiabetic,” “antimicrobial,” “obesity,” “cardiovascular diseases,” “neurodegenerative diseases,” and “health effects.” Boolean operators (“and,” “or”) were used to refine the search strategy. Only articles published in English were considered, while preprints, conference abstracts without full text, and non-peer-reviewed sources were excluded.

Initially, all retrieved studies were screened based on titles and abstracts. Full-text articles of potentially relevant studies were then assessed for eligibility. Studies not directly related to bromelain’s biological activities or health effects were excluded. Additionally, the reference lists of the selected articles were manually screened to identify further relevant studies. The review included *in vitro, in vivo*, animal, and human studies, as well as clinical trials, systematic reviews, and meta-analyses. All eligible studies were evaluated in terms of study design, methodology, and reported outcomes, and the findings were synthesized to provide a comprehensive overview of the current literature.

## 3 Chemical structure and bioactive properties of bromelain

Bromelain is a protease obtained via extraction from the fruit or stem of pineapple (*Ananas comosus* (L.) Merr.) (8). It constitutes a group of enzymes capable of catalyzing proteolytic reactions to break down proteins into smaller polypeptides or single amino acids. More specifically, it is classified as a cysteine proteinase based on the cysteine thiol in its active site (9). Various cysteine endopeptidases, peroxidases, phosphatases, ribonucleases, protease inhibitors, carbohydrates, cellulases, and glycoproteins are found in raw bromelain, as well as organically bound calcium (6, 9–12). A sulfhydryl moiety shapes its functional element (12).

The secondary structure of stem bromelain is highly stable; it maintains its activity between pH 7 and 10; however, activity declines outside this range, and it becomes irreversibly inactivated at pH levels above 10, reflecting the sensitivity of its catalytic cysteine residue to extreme pH conditions (13–15). Furthermore, bromelain remains stable for extended periods of time when stored at −20 °C (12, 13). The thermal stability of most enzymes decreases with increasing temperature, and enzymatic activities are seriously inhibited above 65 °C. However, bromelain shows thermal stability in the course of processes that involve heating up to 60 °C during its extraction stage from pineapple plants (16). Due to its protein structure, bromelain is readily digested after oral intake. The inactivation or inhibition of its biological activities is generally attributed to denaturation in the stomach environment. Therefore, many food supplements containing bromelain are formulated as capsules or enteric-coated tablets (17).

## 4 Antioxidant, anti-inflammatory, immunomodulatory, antinociceptive, and antithrombotic properties and effects of bromelain

Bromelain, as a protease enzyme, exerts antioxidant activities while playing multiple roles in free radical scavenging and lipid peroxidation inhibition (18). It is classified as a cysteine protease that hydrolyzes peptide bonds in proteins through a catalytic cysteine residue at its active site, rather than degrading all proteins containing cysteine. It indirectly prevents the biosynthesis of proinflammatory prostaglandins, leading to the reduction of inflammatory symptoms (15, 19). In addition to these properties, bromelain has attracted increasing attention for its analgesic and immune-supporting effects, with a growing body of literature highlighting its immunomodulatory and antinociceptive activities.

### 4.1 Antioxidant effects of bromelain

Pineapple contains various antioxidant compounds, including vitamins, flavonoids, and phenolic compounds, which contribute to its overall antioxidant capacity (20, 21). However, these components are distinct from bromelain, a proteolytic enzyme complex whose antioxidant effects are mediated through different biological mechanisms. Bromelain has been identified as an antioxidant primarily through its ability to modulate endogenous antioxidant defense systems. It enhances endogenous antioxidant defense by increasing the activity of key antioxidant enzymes, including catalase and superoxide dismutase, and by elevating glutathione levels, partly through the upregulation of transcription factors such as Nrf1 and Nrf2. Mechanistically, bromelain activates the Nrf2 signaling pathway, leading to transcriptional upregulation of antioxidant enzymes such as catalase, superoxide dismutase, and glutathione-related enzymes, thereby enhancing cellular resilience to oxidative stress (22). In addition, bromelain has been reported to reduce the expression of inducible nitric oxide synthase (iNOS), thereby decreasing the production of reactive nitrogen species and nitric oxide under inflammatory conditions (23). The antioxidant activity of bromelain has also been shown to be dose-dependent, with higher concentrations producing more rapid and pronounced effects (24). At lower concentrations, the antioxidant effect is less immediate but may increase gradually over time, suggesting a time-dependent enhancement of antioxidant capacity (17). This distinction indicates that bromelain exhibits both dose- and time-dependent antioxidant properties. In addition, bromelain’s biological effects, including inhibition of platelet aggregation, modulation of inflammatory responses, and facilitation of skin debridement, have been associated with its capacity to influence oxidative stress and tissue repair processes (4). Supporting these findings, animal studies have demonstrated increased levels of antioxidant enzymes such as glutathione-S-transferase, glutathione peroxidase, and superoxide dismutase following bromelain supplementation (24). Thus, the antioxidant effects observed in studies using whole pineapple extracts should not be solely attributed to bromelain.

### 4.2 Anti-inflammatory effects of bromelain

Another important feature of bromelain is its ability to exert anti-inflammatory effects. The most common application areas in this regard include the treatment of inflammatory edema in surgical and medical fields. Bromelain decreases inflammation and edema when administered intraperitoneally or orally at doses of 5–10 mg/kg in animals, reducing inflammation and edema due to histamine, carrageenan, dextran, egg albumin, and formalin (25). Bromelain inhibits the production of serotonin and bradykinin in inflamed tissues and impacts capillary permeability, helping to decrease vasodilation, leukocyte infiltration, and localized pain (12). In one study, it was demonstrated that bromelain was particularly effective against superoxide radicals and showed antioxidant and anti-inflammatory properties by inhibiting phosphorylated- mitogen-activated protein kinase (MAPK) signaling pathways (26). Furthermore, crude and purified rhizome bromelains reduced the levels of proinflammatory cytokines induced by lipopolysaccharide-induced inflammation in RAW 264.7 macrophage cells and inhibited MAPK and nuclear factor kappa B (NF-κB) signaling pathways, with purified bromelain being a more potent inhibitor than crude bromelain (27). Bromelain has also been reported to modulate immune responses rather than exerting purely suppressive effects. While it can reduce helper T-cell activation, it may differentially regulate cytokine production depending on the cellular context and inflammatory status. For instance, bromelain has been shown to influence cytokines such as interleukin (IL)-2, IL-6, and granulocyte-macrophage colony-stimulating factor, reflecting its immunomodulatory rather than strictly pro- or anti-inflammatory role (28). Consequently, bromelain can achieve significant anti-inflammatory effects across diverse pathological conditions. At the molecular level, bromelain exerts anti-inflammatory effects primarily by inhibiting NF-κB nuclear translocation through stabilization of IκBα, suppressing phosphorylation of MAPK pathway components (ERK, JNK, and p38), and reducing the transcription of pro-inflammatory cytokines such as TNF-α and IL-6.

### 4.3 Immunomodulatory effects of bromelain

The immunomodulatory effects of bromelain are remarkable because this enzyme is capable of both activating and suppressing the immune system. Bromelain increases the activation of cluster of differentiation 2-mediated T cells and the independent binding of T cells to the spleen, and it increases the development of interferon-γ-dependent TNF-α, IL-1α, and IL-6 in human peripheral blood mononuclear cells (12, 29). Bromelain has been reported to exert bidirectional immunomodulatory effects, whereby it can either stimulate or suppress T-cell responses depending on factors such as cell type, immune status, and concentration. This bidirectional effect is mediated through context-dependent modulation of immune signaling pathways, including NF-κB and cytokine-regulated networks, allowing bromelain to maintain immune homeostasis rather than exerting purely stimulatory or suppressive effects (15). As demonstrated by Tallei et al. (30), the carbohydrate portion of bromelain is a strong immunomodulator and exerts its highest binding energy for the IκBα/NF-κB p65 homodimer complex, IL-1β, IL-6, and TNF-α, respectively. Their findings indicate that the carbohydrate portion of bromelain may be a potential lead molecule for future drugs developed to avoid or treat inflammation and ensure the maintenance of balance in the immune system.

### 4.4 Antinociceptive effects of bromelain

Bromelain has analgesic effects and is widely used in treatments for muscle and perineal pain, arthritis, and episiotomy pain (23). The analgesic/antinociceptive properties of bromelain in the modulation of neuropathic pain pathways have been shown to reduce hyperalgesia and allodynia with 21 days of treatment after chronic compression injury (31). Another study involving the use of bromelain in the management of chronic compression injury revealed that bromelain protects the neuronal electrolyte balance (32).

### 4.5 Antithrombotic effects of bromelain

Bromelain demonstrates inhibitory activity against both intrinsic and extrinsic coagulation pathway activation in addition to prolonging prothrombin and activated partial thromboplastin times; thus, it prevents clot formation. However, current evidence indicates that bromelain does not exert a strong direct anticoagulant effect; rather, its influence on hemostasis is primarily indirect and context-dependent. Bromelain extends blood clotting time by enhancing fibrinolysis without significantly affecting fibrinogen levels (33). In addition to its fibrinolytic activity, bromelain has been shown to inhibit platelet aggregation, thereby contributing to its antithrombotic effects (34, 35). These effects are thought to be mediated through the proteolytic modulation of key proteins involved in coagulation and platelet function, rather than direct inhibition of coagulation factors. In addition, it modulates blood coagulation by selectively modulating the levels of prostaglandin E2 (PGE2) and thromboxane A2 (TXA2) (34). Bromelain achieves this effect in a dose-dependent manner by causing decreases in the levels of PGE2 and TXA2 activity and by changing the TXA2/prostacyclin (PGI2) ratio in favor of the anti-inflammatory PGI2 (35). In an experimental murine model of non-alcoholic fatty liver disease, chronic administration of 20 mg/kg bromelain daily for 12 weeks yielded significant antithrombotic effects, as evidenced by reduced hepatic thrombus formation and normalization of high-fat diet-induced coagulation parameters. Specifically, the intervention restored the previously shortened prothrombin time, activated fibrinogen time, and partial thromboplastin time while simultaneously elevating plasminogen levels and attenuating factor XIII expression according to measurements performed for both hepatic and plasma specimens (36). However, these findings are derived from animal models using mg/kg dosing and may not be directly translatable to human clinical conditions. Overall, bromelain appears to regulate coagulation through multiple interconnected mechanisms, including fibrinolysis, platelet aggregation inhibition, and modulation of inflammatory mediators; nevertheless, current clinical evidence remains limited, and its anticoagulant or antithrombotic efficacy in humans has not yet been conclusively established. Taken together, these findings suggest that bromelain does not act as a classical anticoagulant but rather modulates hemostasis indirectly through fibrinolytic and antiplatelet mechanisms, which may vary depending on dose, formulation, and experimental context.

## 5 Role of bromelain in disease management

Bromelain has diverse effects that allow for its wide use as a phytotherapeutic agent (37, 38). Some *in vitro, in vivo*, animal, human, and clinical studies investigating the effects of bromelain on health are summarized in Table 1.

**Table 1:** Some *in vitro*, *in vivo*, cell line, animal, human, and clinical studies on the effects of bromelain on health.

Study design	Dose	Duration	Effects	References
40 rats 5 groups	1,000 mg/kg/day bromelain 800 mg/kg/day papain	3 weeks	Reduced symptoms of intestinal inflammation; increased biomarkers of oxidative stress and pro-inflammatory cytokines	(94)
24 rats Neurotoxin 6-OHDA injection	Bromelain (40 mg/kg intraperitoneally)	Before or 24 h after the 6-OHDA lesion	Decrease in the plasma concentration of neutrophils and platelets and in the plasma concentration of TNF-α and IL-1β	(95)
66 albino Wistar rats 11 groups	20–40 mg/kg bromelain	–	Complications of interstitial pneumonia and fibrinous bronchopneumonia in lung tissue were reduced	(99)
Sixty-four male Wistar rats randomly divided into 8 groups	30 mg/kg or 50 mg/kg bromelain	21 days	Improvement in neuropathic pain; inhibition of anxiety-like behaviors; attenuated increases in cerebral cortex IL-1β, IL-6, and PGE2 levels; decreases in NF-κB, IL-1β, IL-6, TNF-α, PGE2, and nitrate concentrations and iNOS expression in the sciatic nerve	(100)
28 male adult Wistar rats	Bromelain (250 mg/kg) and AlCl_3_ (34 mg/kg, 1/25 LD_50_)	1 month	Bromelain supplementation before aluminum poisoning in rats improved oxidative stress markers, hormone levels, and sperm quality compared to group receiving only aluminum; TBARS and H_2_O_2_ levels decreased only in rats receiving bromelain supplementation	(101)
40 patients with knee osteoarthritis	Oral bromelain (500 mg/day) or diclofenac (100 mg/day)	16 weeks	Improvement in total WOMAC scores and pain subscales, stiffness subscales, and function subscales and the physical component of the SF-36; reduction in PGE2 production compared to baseline values	(102)
42 patients requiring extraction of a single mandibular third molar under local anesthesia	Oral Brome-Inf®, purified bromelain Brome-Inf^®^ (freeze-dried pineapple powder containing 200 mg of bromelain in every 2.5 g of powder; 200 mg of bromelain 2,500 GDU/g in every 2.5 g of powder)	7 days	Higher ibuprofen requirement in the placebo group; reductions in pain and swelling were significantly higher in both the bromelain and pineapple groups than in the placebo group; both bromelain and Brome-Inf® supplementation reduced the need for ibuprofen	(103)
20 patients with mucinous tumors	20–60 mg bromelain	24 h	Objective response to treatment and significant mucolytic activity according to radiological appearance observed in 73.2% of treated sites	(104)
68 diabetic patients	Bromelain at 3 × 350 mg (1,050 mg/day)	12 weeks	There was a change in fibrinogen level in the bromelain group	(105)
28 mice	25 mg/kg bromelain	28 days	Reduction in tumor size and lung metastasis; tumor inflammation genes (gremlin (GREM1), IL-1β, IL-4, NF-κB1 and prostaglandin-endoperoxide synthase 2 (PTGS2)), tumor nitric oxide levels, and serum IL-1β and IL-4 levels were reduced	(87)
Lipopolysaccharide-induced human dental pulp cells	2.5, 5, 10, 20 μg/mL bromelain 5 μg/mL LPS	–	Decreased levels of IL-1β, IL-6, IL-8, ICAM-1, and VCAM-1 and decreased phosphorylation of p65 in the cytoplasm and nucleus	(106)
Bacterial nanocellulose membranes	10 mg/mL bromelain	–	Antimicrobial effects against gram-positive bacteria in wound healing	(107)
30 burn patients	NexoBrid®, containing proteolytic enzymes enriched in bromelain	–	Bromelain was a viable alternative to surgical debridement, providing speed, tissue selectivity, safety, and less blood loss	(108)

### 5.1 Bromelain and its antiulcer effects

Gastric ulcer is a multifactorial disease resulting from an imbalance between aggressive factors, such as gastric acid, pepsin, *Helicobacter pylori* infection, and nonsteroidal anti-inflammatory drugs (NSAIDs), and protective mechanisms including the mucosal barrier, antioxidant defense systems, and adequate mucosal blood flow. Disruption of the gastric mucosal barrier increased oxidative stress, and inflammation are key contributors to ulcer development (39). In albino rats, bromelain exhibited significant dose-dependent ulcer-protective effects in both pylorus ligation and ulcer models. Bromelain treatment led to decreases in free and total acidity, which were accompanied by a corresponding increase in gastric pH, indicating reduced gastric acid secretion and improved mucosal protection. Additionally, ulcer index values were significantly reduced in treated animals (40). In another study, administration of 50 mg/kg bromelain demonstrated marked antiulcer effects in indomethacin-induced gastric ulcer models. These effects were associated with reduced gastric tissue myeloperoxidase activity and enhanced antioxidant defense, as evidenced by increased levels of catalase, paraoxonase, glutathione peroxidase, and arylesterase. Furthermore, bromelain exerted anti-inflammatory effects by upregulating heme oxygenase-1 and Nrf2 expression while reducing IL-33 levels (41).

### 5.2 Bromelain and its anti-obesity/antihyperlipidemic effects

Bromelain is among the known phytotherapeutic compounds showing anti-obesity properties (42). In an experimental study, mice receiving high-fat diets for 12 weeks showed approximately 30% increase in body weight. However, in mice that were given bromelain supplementation at 20 mg/kg together with a high-fat diet, the weight of the liver and other organs decreased by approximately 20% (43). In a human study, Hasoon et al. (44) reported that individuals receiving bromelain supplementation exhibited decreases in both body mass index and waist circumference measurements relative to control subjects, while waist-to-hip ratio remained statistically unchanged between the groups.

Stem bromelain, a phytotherapy protein, is considered an alternative approach in obesity treatment. An *in vitro* study revealed that stem bromelain suppressed the expression of adipogenic genes in 3T3-L1 adipocytes while inducing apoptosis and lipolysis in mature fat cells. It suppressed adipogenesis by downregulating CCAAT/enhancer-binding protein (C/EBP) and PPAR-γ expression independently of C/EBP gene activity. Furthermore, adipose tissue dysfunction associated with obesity leads to increased release of actin and proinflammatory cytokines, contributing to the development of insulin resistance (45).

One proposed mechanism involves inhibition of the Akt signaling pathway, which plays a central role in adipocyte survival and differentiation. Suppression of Akt activity may promote apoptosis and reduce adipocyte proliferation, thereby contributing to reduced adipose tissue accumulation (44). Additional investigations revealed that the administration of pineapple leaf extract and dried pineapple fragments resulted in significant body weight reduction in hypercholesterolemic animal models. These effects may not be exclusively attributed to bromelain, as whole pineapple extracts contain multiple bioactive compounds that may contribute synergistically (46).

Animals with higher levels of brown adipose tissue were shown to exhibit greater resistance to obesity and type 2 diabetes, as human brown adipose tissue, like that of mice, enhances fat oxidation and overall energy expenditure (47). Obesity has been linked to reduced brown adipose tissue function and activity, and both obesity and type 2 diabetes are linked to reduced glucose uptake in brown adipose tissue (48). Therefore, bromelain may contribute to metabolic regulation and energy balance; however, its effects should be interpreted within the context of complex metabolic pathways rather than as a standalone anti-obesity solution. Recent research indicates that bromelain supplementation may help reduce obesity, enhance insulin sensitivity, and exert anti-inflammatory effects in individuals with obesity and diabetes (44).

Lipid imbalances in cases of obesity entail elevated serum triglycerides, cholesterol, apolipoprotein B, and low-density lipoprotein cholesterol levels, while high-density lipoprotein cholesterol levels may also be affected (49). Bromelain has been reported to exert antihyperlipidemic effects, including reductions in cholesterol levels, although the underlying mechanisms remain under investigation (29). Although bromelain has various applications in pharmaceutical and medical fields, challenges arise in the course of its use due to the limited stability of enzymes and their susceptibility to denaturation and modifications that can reduce their activities. Through new nanotechnological methods, it is possible to enhance bromelain’s enzymatic activity and properties, thus producing better formulations (15).

### 5.3 Bromelain and its relationship with cancer

Bromelain demonstrates anticancer potential through its ability to activate various cellular death pathways, including apoptotic, necrotic, and autophagic mechanisms (50). Importantly, bromelain’s antioxidant and anti-inflammatory properties are not associated with reduced apoptosis in cancer cells; rather, these effects contribute to the modulation of tumor-associated inflammation and oxidative stress, thereby supporting its overall anticancer activity (51). Research has revealed several mechanisms through which bromelain affects cancer cells, including the inhibition of cancer cell growth, induction of apoptosis, modulation of inflammatory pathways, exertion of antiangiogenic effects, enhancement of immune function, and stimulation of sensitivity to treatment in cancer cells (50, 52).

A recent study explored a therapeutic approach combining bromelain with acetylcysteine (BromAc), suggesting potential synergistic effects in cancer-related applications (53). However, these findings are primarily derived from preclinical and experimental studies, and their clinical relevance remains to be fully established. One of bromelain’s key anticancer properties arises from its ability to induce apoptosis, thereby suppressing cancer cell proliferation (50). Mechanistically, bromelain has been shown to influence key regulatory pathways such as Akt signaling, which plays a central role in cell survival and apoptosis regulation (28).

Different concentrations of bromelain have been reported to induce cytotoxic effects in cancer cells, particularly in *in vitro* models such as human cancer cell lines (54). In colorectal cancer cell lines, bromelain treatment increased reactive oxygen species production and autophagy, leading to reduced proliferation capacity (55). In addition, bromelain has been shown to induce iron-dependent regulated cell death mechanisms, including ferroptosis, which is characterized by lipid peroxidation and contributes to cancer cell destruction (56). These findings suggest that bromelain may exert anticancer effects through multiple, complementary cell death pathways (57, 58).

Another *in vivo* study concluded that bromelain and peroxidase increased the levels of hemoglobin and white blood cells in mice with Dalton’s lymphoma. Apoptosis was also induced through the downregulation of Bcl-2 and NF-κβ (59). Bromelain administration in C57BL/6 N mice was found to reduce tissue necrosis by 25%. Compared to controls, greater density of functional microvessels was achieved, and decreases in myeloperoxidase-positive neutrophils and apoptotic cells were observed (60). Therefore, bromelain could potentially be used to prevent cell necrosis in the future. Additionally, bromelain’s prominent analgesic properties are based on its ability to act directly on pain mediators such as bradykinin. These properties, together with the lack of major side effects reported even after long-term use, make bromelain promising from an oncological perspective (28).

These findings indicate that bromelain may suppress cancer cell growth and enhance the efficacy of conventional therapies. However, current evidence is predominantly based on *in vitro* and *in vivo* studies, and caution should be exercised when extrapolating these findings to humans. Further well-designed clinical studies are required to clarify its therapeutic potential.

### 5.4 Bromelain and its relationship with diabetes mellitus

Diabetes, affecting over 415 million people globally, is a chronic metabolic condition characterized by hyperglycemia, oxidative stress, and inflammation (61). Researchers have demonstrated that daily supplementation of bromelain at 10 mg/kg for 15 days produced multiple beneficial effects in rats; it lowered hepatic malondialdehyde, cholesterol, serum triglycerides, and fasting blood glucose while simultaneously increasing total protein levels, serum albumin, and wound healing rates. Additionally, bromelain was found to counteract streptozotocin-induced increases in several hepatic markers, including lysophosphatidic acid levels, oxidized low-density lipoprotein, and the expression of lysophosphatidic acid receptor 1 and beta-secretase proteins in tissue (62). From a metabolic perspective, bromelain supplementation has been associated with improvements in insulin resistance and inflammatory markers. In a human study, Hasoon et al. (44) reported that bromelain supplementation at 1,000 mg/day reduced serum leptin, HOMA-IR, TNF-α, and IL-6 levels in individuals with type 2 diabetes.

In addition to its metabolic effects, bromelain has been shown to contribute to wound healing processes in diabetic conditions. One study demonstrated that bromelain was effective in healing diabetes-related wounds in rats with type 1 diabetes mellitus; it reduced granulation tissue formation, macrophage numbers, and epithelial thickness after 1 week while promoting wound shrinkage and increasing the numbers of neovessels (63). Similarly, bromelain has been reported to enhance wound healing in diabetic conditions by reducing inflammation through the downregulation of MAPK/ERK and NF-κB signaling pathways at concentrations of 2.5–20 mg/mL (64).

### 5.5 Bromelain and its relationships with neurodegenerative diseases

Neurodegenerative diseases are fundamentally characterized by inflammation and oxidative stress, both of which are contributors to neuronal apoptosis and progressive neuronal damage. Persistent oxidative stress and chronic inflammation can exacerbate neuronal injuries by triggering apoptotic pathways (25). These diseases, including dementia, Alzheimer’s disease, and Parkinson’s disease, constitute a major societal burden, particularly affecting the elderly population (65).

Bromelain, as a result of its potent antioxidant and anti-inflammatory properties, may have a protective role in ameliorating the pathological processes occurring in these diseases (21, 66). The majority of currently available medications for neurodegenerative disorders offer only transient symptomatic relief rather than halting disease progression (8, 32, 67). Bromelain exerts immunomodulatory effects by regulating T-cell and macrophage activity and by preventing leukocyte migration to inflamed neural tissues (68). It has also been shown to attenuate proinflammatory signaling by modulating microglial activation (68) and to protect neuronal cells against oxidative injury through the neutralization of free radicals and reactive oxygen species (69).

The anti-inflammatory potential of bromelain involves the downregulation of proinflammatory cytokines and the modulation of microglial activity. Moreover, its antioxidant capacity contributes to reduced oxidative stress by scavenging free radicals, inhibiting the production of reactive oxygen species, and chelating redox-active metal ions (27). Together, these mechanisms support neuronal survival and help limit apoptotic cell death.

The dysregulation of immune function is closely linked to the onset and advancement of neurodegenerative diseases (70). Microglial cells, for example, are closely associated with amyloid plaques in Alzheimer’s disease (71). Bromelain can regulate immune-related gene expression by suppressing TNF-α-induced MAPK and NF-κB signaling cascades (72). In experimental autoimmune models, bromelain was administered as part of a Phlogenzym formulation containing rutin and trypsin and it demonstrated neuroprotective and immunomodulatory potential in multiple sclerosis models, possibly by modulating T-cell activation and antigen-presenting cell interactions (73).

**Figure 1:**
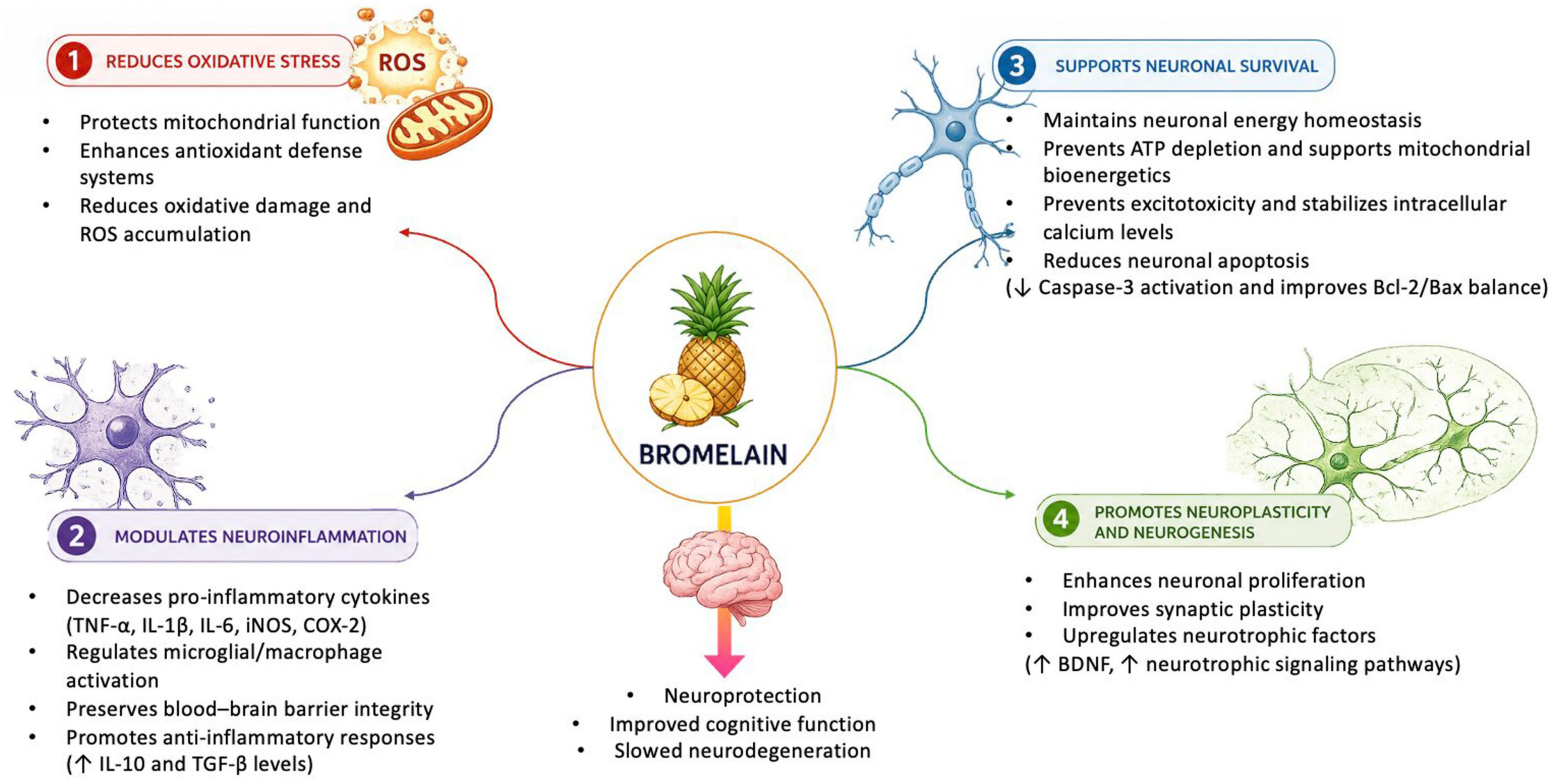
The potential neuroprotective mechanisms of bromelain on neurodegenerative diseases (78).

Additionally, bromelain exhibits neuroprotective effects by inhibiting the aggregation of β-amyloid and α-synuclein proteins. The accumulation of β-amyloid increases oxidative stress and intracellular Ca^2+^ influx, promoting neuronal toxicity in cases of Alzheimer’s disease (66). Bromelain may prevent β-amyloid peptide aggregation, thereby reducing amyloid plaque formation (74). Although studies on α-synuclein aggregation are limited, some evidence suggests that structural similarities between bromelain and α-synuclein may influence synucleinopathy pathways (73). Furthermore, bromelain may modulate tau protein phosphorylation, potentially reducing neurofibrillary tangle formation and related pathological alterations (75). However, the proposed effects of bromelain on α-synuclein aggregation and tau phosphorylation are currently based on limited and indirect evidence, primarily derived from *in vitro* studies on other natural compounds and computational or theoretical approaches, and should therefore be considered hypothetical, as direct *in vitro* or *in vivo* experimental data specific to bromelain are lacking. Similar mechanisms involving natural compounds in neurodegenerative disease models have been reported (76, 77). Therefore, these proposed mechanisms should be interpreted cautiously, as direct experimental evidence specific to bromelain remains limited. The potential neuroprotective mechanisms of bromelain in cases of neurodegenerative diseases are schematically presented in Figure 1 (78).

### 5.6 Bromelain and its relationship with infection

Infections may present with a range of systemic symptoms, including gastrointestinal and inflammatory manifestations (79). Bromelain has been investigated for its potential antiviral and antimicrobial effects, primarily through its proteolytic activity and immunomodulatory properties. Viral infections involve the interaction of viral proteins with host cell receptors, followed by replication within host cells. In this context, proteolytic enzymes such as bromelain may interfere with viral entry and replication processes by modifying viral or host proteins (80).

The zinc metallophosphatase domain of ACE2, which is the primary receptor for viral entry into the host, binds to the viral spike protein S. Both the ACE2 receptor and transmembrane protease serine 2 (TMPRSS29), which are, respectively, necessary for viral entry and spike protein activation, are enriched with cysteine residues. ACE-2 has been found to possess 6 cysteine residues with 3 disulfide bonds for stabilization, while TMPRSS2 is estimated to entail 18 cysteine residues with 9 disulfide bonds for stabilization (81). In an *in vitro* study conducted by Sagar et al. (82) using the Vero E6 cell line, bromelain was shown to interact with ACE2 and TMPRSS2 and reduce spike protein expression in a dose-dependent manner; however, these findings are preliminary and limited to experimental models (82). According to another study, the combined application of bromelain and curcumin helped to prevent infection by enhancing the human immune response (83).

Although bromelain has shown potential to interfere with viral entry mechanisms, current evidence is largely based on *in vitro* and preclinical studies, and its clinical efficacy in infection treatment remains unclear. Bromelain can be consumed by adding pineapple to one’s daily diet or by taking a concentrated supplement (82). Therefore, bromelain should be considered a supportive or complementary agent rather than a primary treatment, and further well-designed experimental and clinical studies are required to confirm its therapeutic potential.

## 6 Effects of bromelain supplementation

Bromelain, a proteolytic enzyme complex derived from *Ananas comosus*, has gained increasing attention as a dietary supplement due to its potential therapeutic properties and relatively favorable safety profile. It has been reported to exhibit measurable systemic absorption following oral administration, despite the partial inactivation of proteolytic enzymes in the gastrointestinal tract (83, 84). Its bioavailability has been supported by clinical evidence demonstrating the presence of proteolytically active bromelain in serum after oral intake, indicating that a fraction of the enzyme remains intact and biologically active (85). Emerging evidence suggests that bromelain may exert beneficial effects in metabolic disorders and cancer; however, these findings are predominantly based on *in vitro* and animal studies, with limited evidence from human clinical trials (28, 86). It has been found to make beneficial contributions in the treatment of diverse ailments including various inflammatory cardiovascular diseases and sports injuries. In both preclinical and clinical studies, bromelain supplements were found to have antimicrobial, anti-inflammatory, antitumoral, and immunomodulatory effects (14, 87, 88). Considering the data available in the literature, it can be said that bromelain supplementation offers great promise in the field of health in terms of its functional properties and mechanisms of action (89).

According to Commission E of Germany’s Federal Institute for Drugs and Medical Devices, the recommended daily dose of bromelain is 80–320 mg and it should be taken two or three times a day. However, bromelain should be used with caution as it has a blood-thinning effect and delays clotting. Furthermore, those who are allergic to pineapple should not use it. Other dose recommendations are 500 mg with food in divided doses to aid digestion, 500 mg four times a day on an empty stomach for injuries, and 500–2,000 mg twice a day for arthritis treatment. Bromelain is primarily available as oral formulations, including tablets and capsules, but may also be applied topically in the form of creams. Its use is typically recommended for short durations, generally not exceeding 10 days (90–96).

Evidence from human studies indicates that bromelain supplementation has been applied across a wide range of doses, varying according to the clinical context and study design. Data from randomized controlled trials show that bromelain doses range between approximately 99.9 and 1,200 mg/day when used in combination therapies, and between 200 and 1,050 mg/day when administered alone, with intervention periods spanning from 1 week to 16 weeks (97). In line with these findings, other studies report a similarly broad dosing range, generally between 100 and 1,200 mg/day (70). This considerable variability in both dosage and duration may partly explain the inconsistencies observed across clinical outcomes and highlights the need for more standardized dosing approaches supported by well-designed clinical trials. The variability in dosing regimens across studies, including differences in units (mg/kg in animal studies versus mg/day in human studies), complicates direct comparison and highlights the need for standardized clinical dosing guidelines.

Bromelain supplementation should be supervised by physicians, as it may increase the risk of bleeding when used concomitantly with anticoagulant or antiplatelet agents such as aspirin, warfarin, heparin, or NSAIDs (93). Although bromelain has not demonstrated significant direct anticoagulant effects when administered alone, evidence suggests that it may influence hemostasis indirectly through inhibition of platelet aggregation and enhancement of fibrinolytic activity (15, 94, 98). However, current clinical evidence remains limited and inconsistent, and a clear anticoagulant effect in humans has not been firmly established. Consistent with this, one study reported that bromelain alone did not significantly alter coagulation parameters, whereas its combination with N-acetylcysteine significantly prolonged bleeding time and affected coagulation markers (94). Therefore, the apparent inconsistency in its anticoagulant effects may be explained by its indirect and context-dependent actions, particularly when combined with other agents affecting coagulation.

## 7 Side effects of bromelain

Reported adverse effects of bromelain in human studies are generally mild and primarily gastrointestinal in nature. In a systematic review of randomized controlled trials, a limited number of participants experienced adverse events, most of which were gastrointestinal, and only a small proportion discontinued treatment, indicating good overall tolerability (97). Other symptoms such as nausea, vomiting, and diarrhea have also been reported in the literature (15).

However, some additional risks, including a potential increase in bleeding tendency, have been suggested based on theoretical mechanisms or limited evidence rather than consistent clinical findings (15). Bromelain has been shown to modulate hemostatic processes through inhibition of platelet aggregation and enhancement of fibrinolytic activity, which may contribute to an increased bleeding risk, particularly when combined with anticoagulant or antiplatelet agents (70, 86). Similarly, concerns regarding uterine stimulation have been raised, although these are largely precautionary and not supported by robust clinical data (15). Therefore, bromelain use is generally not recommended in individuals with bleeding disorders or during pregnancy and lactation as a precautionary measure.

## 8 Conclusion

Bromelain is a complex bioactive mixture primarily composed of proteolytic enzymes, with additional non-proteolytic components such as glycoproteins and other minor constituents. Its reported anti-inflammatory, antioxidant, immunomodulatory, anticancer, and antiulcer properties highlight its potential relevance in various disease contexts, including metabolic, cardiovascular, and neurodegenerative disorders.

Current evidence suggests that bromelain may exert beneficial effects through modulation of inflammatory pathways, reduction of oxidative stress, and regulation of immune responses, which may play roles in the pathophysiology of neurodegenerative diseases, cancer, obesity, hyperlipidemia, infection, and other conditions (78). Overall, bromelain appears to exert its biological effects through a multi-target mechanism involving suppression of pro-inflammatory pathways (NF-κB, MAPK), activation of antioxidant responses (Nrf2), modulation of immune signaling, and proteolytic regulation of key molecular targets. These interconnected mechanisms may explain its broad therapeutic potential across diverse disease conditions. In addition, its potential roles in adipogenesis, lipid metabolism, and neuronal protection have attracted increasing research interest. However, despite these promising findings, most of the available data are derived from *in vitro* and preclinical studies.

Therefore, there is a clear need for well-designed clinical trials to establish its efficacy, optimal dosing, and long-term safety in human populations. Furthermore, variability in enzyme composition and the lack of standardized formulations remain important challenges that limit its clinical applicability. Future research should focus on clinical validation, dose optimization, and the development of effective delivery systems to improve stability and bioavailability. Addressing these limitations will be essential for translating bromelain into safe and effective therapeutic applications. Importantly, distinguishing the specific effects of bromelain from those of other pineapple-derived bioactive compounds remains a critical challenge in the interpretation of current evidence.

## 9 Recommendations and future perspectives

Future research should prioritize well-designed clinical trials to establish the efficacy, optimal dosing, and long-term safety of bromelain across different disease conditions, as current evidence remains largely limited to preclinical and small-scale studies.

From a formulation perspective, improving the stability and delivery of bromelain remains a critical challenge due to its proteolytic and sensitive nature. In this context, nanotechnology-based delivery systems, such as nanoencapsulation and nanoparticle carriers, have shown promise in enhancing its bioavailability and protecting enzymatic activity; however, further *in vivo* and clinical validation studies are needed. Another important area for future investigation is the exploration of bromelain’s mechanisms of action across different pathological conditions, particularly its interactions with key signaling pathways (e.g., NF-κB, MAPK, and Nrf2) and its regulatory effects on pro-inflammatory cytokines.

Furthermore, significant knowledge gaps remain regarding the standardization of bromelain preparations, variability in enzyme composition, and dose–response relationships, which currently limit its clinical applicability. Finally, in line with growing sustainability concerns, the utilization of pineapple by-products (such as stems and peels) represents a promising strategy for the cost-effective and environmentally sustainable production of bromelain, thereby supporting its broader biomedical and clinical use.

## Glossary

ACE2: Angiotensin converting enzyme 2
ERK: Extracellular signal-regulated kinase
GAE: Gallic Acid Equivalent
HO-1: Heme oxygenase 1
HOMA-IR: Homeostatic Model Assessment of Insulin Resistance
iNOS: Inducible nitric oxide synthase
ICAM-1: Intercellular adhesion molecule-1
VCAM-1: Vascular cell adhesion molecule-1
IL: Interleukin
LPS: Lipopolysaccharide
MAPK: Mitogen-activated protein kinase
NSAIDs: Nonsteroidal anti-inflammatory drugs
Nrf1: Nuclear factor erythroid 2-related factor 1
Nrf2: Nuclear factor erythroid 2-related factor 2
NF-κB: Nuclear factor kappa B
PPAR-γ: Peroxisome proliferator activated receptor gamma
PGI2: Prostacyclin
PGE2: Prostaglandin E2
Akt: Protein kinase B
SF-36: Short Form-36
TXA2: Thromboxane A2
TMPRSS2: Transmembrane protease serine 2
TNF-α: Tumor necrosis factor-alpha
WOMAC: Western Ontario and McMaster Universities Osteoarthritis Index
